# Clinical Research and the Training of Host Country Investigators: Essential Health Priorities for Disease-Endemic Regions

**DOI:** 10.4269/ajtmh.15-0366

**Published:** 2016-02-03

**Authors:** Ousmane A. Koita, Robert L. Murphy, Saharé Fongoro, Boubakar Diallo, Seydou O. Doumbia, Moussa Traoré, Donald J. Krogstad

**Affiliations:** University of the Sciences, Techniques and Technologies of Bamako, Mali; Northwestern University, Chicago, Illinois; Tulane University School of Public Health and Tropical Medicine, New Orleans, Louisiana

## Abstract

The health-care needs and resources of disease-endemic regions such as west Africa have been a major focus during the recent Ebola outbreak. On the basis of that experience, we call attention to two priorities that have unfortunately been ignored thus far: 1) the development of clinical research facilities and 2) the training of host country investigators to ensure that the facilities and expertise necessary to evaluate candidate interventions are available on-site in endemic regions when and where they are needed. In their absence, as illustrated by the recent uncertainty about the use of antivirals and other interventions for Ebola virus disease, the only treatment available may be supportive care, case fatality rates may be unacceptably high and there may be long delays between the time potential interventions become available and it becomes clear whether those interventions are safe or effective. On the basis of our experience in Mali, we urge that the development of clinical research facilities and the training of host country investigators be prioritized in disease-endemic regions such as west Africa.

## Background

As highlighted by the recent Ebola virus disease outbreak,^1^,^2^ impoverished regions with endemic disease such as west Africa are at risk of morbidity, mortality, economic disruption, and civil strife from the epidemic spread of diseases for which prevention and treatment are limited or nonexistent.^3^ The major reasons for this disparity are 1) the diseases that pose the greatest challenge are more frequent in regions with insufficient resources to support drug and vaccine development and few or no opportunities to profit from those investments^4^–^6^ and 2) the numbers of clinical research facilities and trained personnel (clinical investigators, nurses, and laboratory technologists) available to evaluate candidate interventions are severely limited.^6^

The premise underlying this article is that the development of clinical research facilities and the training of host country investigators in endemic regions will accelerate the evaluation of interventions to reduce morbidity and mortality. If these strategies are successful, which we expect they will be, they will relieve a bottleneck in the progression from basic research to implementation (bench to bedside) by decreasing the time required to evaluate candidate interventions. These strategies also have the potential to ensure that a single set of standards for efficacy and safety testing is used across the globe, that is, the standards used to evaluate drugs, vaccines, and other candidate interventions are the same in impoverished disease-endemic regions as in North America, Europe, Australia, and the developed countries of Asia.^7^,^8^

## Rationale

Neither clinical research nor the training of host country investigators to perform clinical research is currently a priority in endemic regions. In addition, health facilities potentially available for clinical research are typically overwhelmed by large numbers of patients seeking care, a situation that is often exacerbated by national health budgets providing less than $20 per person per year for all preventive and curative health services (Guinea, Liberia, Sierra Leone, and others).^9^ As a result, clinical research in disease-endemic regions often begins with small pilot studies performed by investigators with common interests in scientific questions linked to disease control with access to grant funds that can be used to perform the initial pilot studies.

In this case, the initial discussions about clinical research in Mali began in 2004–2006 because phase 1 (pharmacology, safety) studies of a candidate antimalarial drug in healthy subjects in the United States had shown that the compound being studied (AQ-13) was active in vitro against known drug-resistant *Plasmodium falciparum* and as safe as chloroquine in human subjects.^10^,^11^ Based on those discussions, the Faculty of Medicine and Ministry of Health set aside funds to build a clinical research center on the campus of their major teaching hospital (the Hôpital Point G). The rationale for that decision was to perform phase 2 (efficacy) studies of the candidate antimalarial at the new clinical research center as a test case to examine the feasibility and potential value of clinical trials that required intensive inpatient studies at a clinical research facility linked to a Faculty of Medicine within the disease-endemic area.

## Existing Infrastructure

The development of a clinical research center and the training of host country investigators at the University of Bamako occurred in an environment where African and western investigators had worked together for more than two decades, beginning with studies of mosquito vectors led by Touré^12^ and Gwadz.^13^ On the basis of those and other experiences (e.g., at the Medical Research Council Unit in The Gambia), it is reasonable to expect that the development of clinical research facilities and the training of host country investigators in other regions will succeed most frequently at institutions with an established tradition of international collaboration in clinical research.

## Building and Activating the Clinical Research Center

**Figure 1.**
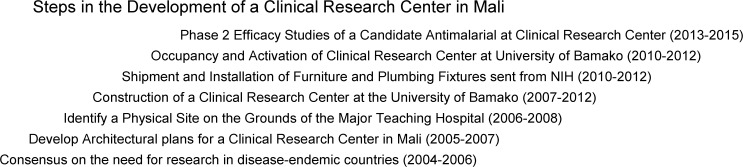
Steps in the development of a clinical research center in Mali. After the initial design and architectural phase (2004–2006), construction of the clinical research center took place from 2007 to 2012. After the installation of plumbing, electricity, and furniture in 2012–2013, the center was occupied in early 2013 and the initial clinical studies of an investigational antimalarial began in July 2013.

**Figure 2.**
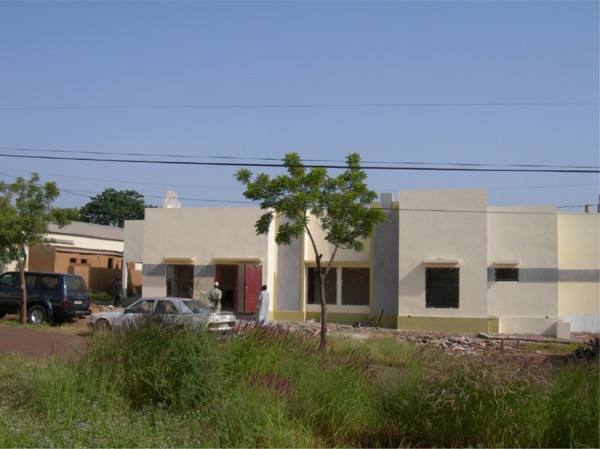
Clinical research center in Mali. The new clinical research center in Mali is on the campus of the major teaching hospital of the University of Bamako (Hôpital Point G). The decision to develop a clinical research center in Mali was based on commitments shared by the Ministry of Health, the Division of Clinical Research (DCR) at National Institute of Allergy and Infectious Diseases (NIAID) and extramural investigators and was based on the anticipated benefits of such a facility for the entire west African region.

During the subsequent 5 years (2007–2012; Figure 1

), the clinical research center was designed and built on the campus of the Hôpital Point G (Figure 2

, Supplemental Table 1) despite delays due to price increases and other challenges. This is because the commitment to develop a clinical research center was shared by the Ministry of Health, the Division of Clinical Research (DCR) at National Institute of Allergy and Infectious Diseases (NIAID), and extramural investigators and was based on its anticipated value for the entire west African region. Subsequently (during 2012 and the first half of 2013), furnishings provided by the DCR were installed, including desks for physicians and nurses; a table and chairs for a conference room; plumbing fixtures (sinks, toilets); and physician's office furniture (examination tables, overhead lights, ophthalmoscopes, and otoscopes). The purposes of this equipment were to facilitate follow-up visits, ophthalmologic examinations, timed venipunctures and finger sticks, the placement of electrodes for Holter recordings, and the computer-based analysis of Holter recordings and other study data.

## Initial Inpatient Studies

After the training of host country investigators and staff (Table 1), 33 subjects with uncomplicated *P. falciparum* malaria (males ≥ 18 years of age) were enrolled in a phase 2 Food and Drug Administration (FDA)–approved efficacy (proof of concept) study to compare an investigational antimalarial (AQ-13) to the currently recommended first-line treatment of uncomplicated *P. falciparum* malaria (Coartem^®^, Novartis, Beijing, China for Novartis, Pharma AG, Basel, Switzerland).^14^ After screening, subjects who provided their informed consent were randomized to either Coartem or the investigational antimalarial, admitted to the clinical research center for 5–6 inpatient days (3 days of treatment and observation plus 2–3 additional days of observation) before discharge on day 6–7, and followed twice weekly as outpatients (weeks 2–6 [days 8–42]) for recurrent parasitemia as evidence of treatment failure. This approach was based in part on previous studies of severe malarial anemia^15^ and has shown that intensive inpatient studies (4 days of 24-hour Holter recordings, 25 timed blood samples on days 1–6 and 24-hour urine collections on days 1–4) can be performed in the disease-endemic area based on patient care and clinical observation provided by host country investigators, pharmacists, and nurses.

## Ebola Testing

The need for rapid diagnostic testing during the 2014 Ebola outbreak^16^ provided an opportunity to use the existing infrastructure in Mali (developed with support from the DCR, the Division of Intramural Research at NIAID/National Institutes of Health and the Centers for Disease Control and Prevention) to provide more rapid diagnosis of Ebola infection. For example, the enhanced biosafety level (BSL) 3 facility developed as part of an International Center of Excellence for Research in Mali was used to ensure that Ebola clinical specimens were inactivated without inadvertent exposure of laboratory personnel. After inactivation under enhanced BSL-3 biocontainment conditions,^17^ those specimens were tested using a two-target reverse transcriptase polymerase chain reaction specific for the polymerase and surface glycoprotein genes of the Ebola virus in a conventional BSL-2 laboratory.^17^–^19^ This strategy permitted the diagnosis of Ebola within 3 hours after specimens were received from the field. As a result, this facility in Bamako became a reference laboratory for affected areas in Mali, because its results were accurate and it reduced the time required to transport specimens to a diagnostic laboratory from 3 days to 2–3 hours.

## Host Country Benefits

### Direct benefits.

The development of a clinical research facility, the training of host country investigators, and the performance of an FDA-approved phase 2 clinical trial described here provide examples of strategies to address the need to evaluate candidate interventions in endemic areas that became clear during the Ebola outbreak.^20^,^21^ Please note that there is reason to expect additional clinical trials of Ebola-related interventions because a candidate vaccine has been shown to be safe and immunogenic in humans,^22^ the postexposure treatment with candidate vaccines may prevent infection and disease,^23^ and a recent study suggests that immunization may reduce human-to-human transmission during an outbreak.^24^ In the short term, the assistance provided by western governments such as the United States, United Kingdom, France, and others has been invaluable. However, in the long-term, the need to evaluate candidate interventions can truly be resolved only by the development of clinical research facilities and laboratories staffed by host country investigators on-site who are knowledgeable about the diseases being studied and have been trained to perform clinical research. Please note that this challenge (bottleneck) is likely to persist for the foreseeable future because investigators in industry and academia are now identifying candidate interventions faster than they can be evaluated by the few clinical research facilities available currently in endemic regions.^21^

### Indirect benefits.

The indirect benefits expected with this strategy include 1) more rapid and more rigorous evaluation of candidate interventions in endemic regions (reduction of the bottleneck described above), 2) improved teaching and patient care in disease-endemic areas provided by host country investigators who themselves are actively involved in clinical research, consistent with the previous experience of developed countries with clinical research centers, 3) better health outcomes in endemic regions from the implementation of interventions found to be effective and the exclusion of interventions found to be ineffective or harmful, and 4) the development of a health research career niche for which the best-prepared individuals will be host country investigators knowledgeable about the diseases being studied who have been trained to perform clinical research.

## Conclusions

On the basis of our experience in west Africa, we are convinced that the development of clinical research facilities and the training of host country investigators can improve health outcomes and the quality of medical and public health practice and teaching in resource-limited regions. We propose a regional rather than country-specific approach because the underlying problems are regional (as exemplified by the recent Ebola outbreak) and because it encourages the careful evaluation of priorities and minimizes infrastructural (overhead) costs. In conclusion, we urge international organizations and funding agencies to support the development of clinical research facilities and the training of host country investigators to facilitate rigorous and more expeditious evaluation of candidate interventions and simultaneously improve the quality of medical and public health practice in resource-limited regions. To maximize the probability of success, we recommend these efforts focus on countries with schools of medicine or public health already engaged in externally funded research on disease control interventions and public health. These essential priorities can no longer be ignored.

## Supplementary Material

Supplemental Table.

## Figures and Tables

**Table 1:** Training of host country investigators and staff

	2004–2006 (planning)	2007–2012* (training)	2013–2015 (phase 2 study)
Mali PI, senior investigators, internal medicine physicians	Review of study protocol and clinical research training in the United States†	Review of study design and the need for detailed written records	Screening, consent, treatment, monitoring for AEs
Consultants in cardiology	Review of the phase 1 studies and rationale for the phase 2 studies	Obtaining and interpreting pre-/post-dose Holter recordings‡	Screening plus follow-up for AEs (especially, arrhythmias and heart block)
Consultant in ophthalmology	Review of phase 1 studies and the phase 2 study design	Screening to identify volunteers with normal ocular function§	Exam to detect ocular AEs based on repeat eye exam 7 days after treatment
Research pharmacist	Review of phase 1 studies and rationale for phase 2 studies§	Review of informed consent, randomization and dosing§	Dosing of individual consented subjects after their randomization
Nursing staff and cardiology technical staff	Review of phase 1 studies and the rationale for the phase 2 studies§	Obtaining timed venous blood samples in relation to dosing§	Written notes each inpatient day focused on results, possible AEs§
Clinical monitor	Assessment of study design for the phase 2 efficacy study†	Prepare operating procedures and bilingual CRFs∥	Daily review of screening, consent, enrollment and other subject records
Laboratory investigators and staff	Test equipment and supplies using appropriate controls	Pilot studies (standardization) based on healthy controls in Mali∥	Hematology and chemistry panels, malaria smears, G6PD testing
Administrative staff	Review administrative/financial issues for the phase 2 studies	Human studies training and certification	Schedule patient transportation, records of patient compensation
Scientific issues	Basis of AQ-13 action against CQ-resistant *Plasmodium falciparum*¶	Safety, pharmacology of AQ-13 in healthy subjects¶	Importance of record keeping, AEs, parasite genotype, treatment failure¶

AEs = adverse events; CQ = chloroquine; CRFs = case report forms; PI = principal investigator.

* Human studies training and certification were required for study personnel in the United States and Mali.

† Training for the Mali PI, senior investigator, and internists was based on two 2-week visits to the United States and on workshops provided by the study PI and the director of the Tulane-LSU Clinical Research Center in New Orleans.

‡ Training on obtaining and interpreting 24-hour Holter recordings was provided to Mali cardiologists by the director of the Tulane Electrophysiology Unit in New Orleans.

§ After approval by the Tulane and Mali Institutional Review Boards and Food and Drug Administration (FDA), the phase 2 study protocol was reviewed with the ophthalmology consultant, research pharmacist, clinical monitor, and other clinical and laboratory staff in Mali by both the PI and the Mali PI. Screening of volunteers for normal vision was performed by an ophthalmology consultant, treatment was provided by a research pharmacist, and timed blood specimens (in relation to dosing) were obtained by the nursing and technical staff.

∥ Bilingual CRFs with numerical or yes/no responses were requested by FDA so study records (including CRFs) could be understood by reviewers who were fluent in English but not in French. Please note that French is used for higher education (including medicine) and official government records in Mali. Standard operating procedures (SOPs) were also prepared in both French and English (bilingual format), although that had not been requested by the FDA Review Panel for the investigational new drug application (IND) under which these studies were performed (IND 055,670).

¶ Scientific issues such as the preparation of SOPs and CRFs, the basis of aminoquinoline action against *P. falciparum* (including the activity of AQ-13 against CQ-resistant parasites), the safety and pharmacokinetics of AQ-13 in healthy human subjects and the molecular basis of parasite genotyping were reviewed with investigators and the technical and nursing staff in Mali by both the PI and Mali PI.
