# Supplementary material for: Clinical Research and the Training of Host Country Investigators: Essential Health Priorities for Disease-Endemic Regions
Source: Am J Trop Med Hyg. 2016 Feb 3;94(2):253–7. doi: 10.4269/ajtmh.15-0366 (PMC4751934; doi:10.4269/ajtmh.15-0366)
Supplement: Supplementary file 1 [file SD1.pdf]

Supplemental Table 1

Steps to developing a clinical research center in Mali

	2004–2006	2007–2012	2013–2015
Rector of the University, dean of the Faculty of Medicine, and the Mali Ministry of Health	Consensus for clinical research on disease control interventions in endemic countries	Site for clinical center on the grounds of teaching hospital, investigative/academic links to university faculty	Support for local purchases, wiring and water lines, participation of faculty as investigators and consultants
Collaborating extramural investigators in the United States	Phase 1 study of candidate antimalarial in the United States	Preliminary studies based on extramural funds	FDA-supported phase 2 efficacy study in Mali
Construction time line	Architectural plans	Build clinical research center	Occupancy, activation
NIH intramural programs, Divisions of Clinical and Intramural Research	Discussion of potential inpatient study protocols	Shipment of furniture and plumbing fixtures	Installation of furniture and plumbing fixtures

FDA = Food and Drug Administration.
